# Vocational factors and mental health during the COVID-19 pandemic in middle-aged and older adults: longitudinal evidence from a population-based cohort

**DOI:** 10.3389/fpubh.2026.1803297

**Published:** 2026-09-11

**Authors:** Till Beutel, Nora Hettich-Damm, Julia Petersen, Markus Schepers, Clemens Köstner, Philipp S. Wild, Rieke Baumkötter, Alica Hartmann, Stephan Letzel, Pavel Dietz, Jan Becker

**Affiliations:** 1 Department of Occupational, Social and Environmental Medicine, University Medical Center of the Johannes Gutenberg-University Mainz, Mainz, Germany; 2 Department of Psychosomatic Medicine and Psychotherapy, University Medical Center of the Johannes Gutenberg-University Mainz, Mainz, Germany; 3 Preventive Cardiology and Preventive Medicine, Center for Cardiology, University Medical Center of the Johannes Gutenberg-University Mainz, Mainz, Germany; 4 Institute of Medical Biostatistics, Epidemiology and Informatics (IMBEI), University Medical Center of the Johannes Gutenberg-University Mainz, Mainz, Germany; 5 Center for Thrombosis and Hemostasis (CTH), University Medical Center of the Johannes Gutenberg-University Mainz, Mainz, Germany; 6 German Center for Cardiovascular Research (DZHK), Partner Site Rhine-Main, Mainz, Germany; 7 Institute for Molecular Biology (IMB), Mainz, Germany; 8 Department of Ophthalmology, University Medical Center of the Johannes Gutenberg-University Mainz, Mainz, Germany

**Keywords:** anxiety, COVID-19, depression, longitudinal study, mental health, occupational health, vocational factors

## Abstract

The COVID-19 pandemic profoundly disrupted public health, social systems, and the workforce. This longitudinal study examines changes in depressive and anxiety symptoms before and during the pandemic in middle-aged and older adults (*N* = 6,813) from the Gutenberg Health and Gutenberg COVID-19 Study in Germany. Using repeated assessments with validated mental health scales (PHQ-9, GAD-2) and accounting for pre-pandemic mental health, we investigated associations with work-related factors (e.g., changes in working hours, remote work, income) and sociodemographic variables (e.g., sex, age, household composition). Average depression and anxiety scores remained relatively stable over time, although the proportion exceeding the clinical PHQ-9 ≥ 10 threshold increased during the pandemic. Pre-pandemic mental health emerged as the strongest predictor of pandemic mental health outcomes. Vocational factors showed only small effect sizes, with increased weekly working hours emerging as the most consistent work-related correlate of distress. Important sociodemographic predictors included female sex, middle age, and living alone. The findings emphasize considering pre-existing mental health and pandemic-related sociodemographic and vocational circumstances when identifying vulnerable groups. Although workplace factors played only a limited role overall, the workplace may nevertheless represent a relevant setting for mental health promotion during future public health crises.

## 1 Introduction

The COVID-19 pandemic and the accompanying public health measures substantially affected social interactions, working conditions, and individual well-being worldwide. Work and employment represent a key link between the pandemic’s macro-level disruptions and individual well-being. During COVID-19, many sectors experienced business closures and mass layoffs (1). Reductions in working hours, job losses, and job insecurity were associated with increased depressive symptoms and anxiety (2–4), while economic hardship emerged as a risk factor for psychological distress and poor mental health outcomes (5–9). Other sectors - particularly healthcare - faced exceptional demands. Increased working hours, higher work intensity, and fear of infection were associated with elevated depressive symptoms, anxiety, and burnout (10–15). Another major workplace change was the large-scale shift toward remote work (16, 17), which offered greater flexibility and reduced exposure risk but also increased social isolation.

Middle-aged and older adults represent a particularly relevant population for studying mental health during the COVID-19 pandemic. Older adults were at higher risk of severe health consequences from COVID-19 and were therefore particularly affected by infection-related concerns and public health restrictions (18, 19). Middle-aged adults, in contrast, often faced the simultaneous demands of employment, financial responsibilities, and caregiving for both children and older family members (20–23). Consequently, mental health trajectories in middle-aged and older populations are likely shaped by a unique combination of health-related, social, and occupational stressors.

Longitudinal evidence suggests that mental health deteriorated during the early stages of the pandemic among middle-aged and older adults, while adaptation and recovery processes were observed over time (24, 25). Importantly, trajectories differed across symptom domains and population subgroups. Anxiety symptoms, for example, tended to recover more rapidly than depressive symptoms, whereas women often experienced more persistent impairment (24, 25). Women were disproportionately affected due to their overrepresentation in sectors with income losses, greater exposure to healthcare-related risks, and heightened caregiving responsibilities, possibly reinforced by persistent gender norms (5, 21–25). Household structure also mattered, as living alone or in separate households increased the risk of social isolation and reduced access to informal support networks during lockdowns, factors that were associated with elevated depressive symptoms during the pandemic (21, 24, 25). Persons with chronic diseases and particularly those with pre-pandemic mental health symptoms were also identified as vulnerable subgroups (22, 24, 25). Overall, longitudinal findings suggest that changes in mental health among middle-aged and older adults were often smaller and more heterogeneous than initially assumed on the basis of early cross-sectional surveys, which frequently portrayed a more uniformly negative picture of the pandemic’s psychological impact (24–27).

Work-related experiences may be especially relevant in middle-aged and older adults because employment often remains a central source of daily structure, social participation, and financial security during later working life. Previous research among middle-aged and older workers suggests that pandemic-related working conditions and employment transitions were associated with mental health outcomes, highlighting the importance of vocational circumstances during later working life (28, 29). For example, workers aged 55 years and older who lost their jobs reported the lowest life satisfaction and the highest levels of loneliness and depressive symptoms. Furloughed workers and those experiencing reductions in working hours or income also showed poorer mental health outcomes than workers whose employment remained unchanged, while working from home was associated with higher anxiety levels (28). These findings highlight the importance of considering vocational factors such as changes in working hours, income changes, home office arrangements, occupational position, and broader changes in work organization when examining psychological well-being in later working life.

However, evidence on the role of specific vocational factors among middle-aged and older adults remains comparatively limited. Much of the existing literature relies on cross-sectional data from specific occupational groups, making it difficult to distinguish pandemic-related associations from pre-existing differences in mental health. Longitudinal studies combining assessments before and during the pandemic within the same participants remain comparatively rare but are essential for examining how vocational and sociodemographic factors are associated with mental health after accounting for pre-pandemic symptom burden. Against this background, the present study uses longitudinal data from a large, population-based cohort of middle-aged and older adults with measurements before and during the pandemic to describe changes in depressive and anxiety symptoms over time. Particular attention is paid to vocational factors among employed participants, including changes in working hours, income changes, home office arrangements, and occupational categories. The aim of the study is to examine whether vocational and sociodemographic factors are associated with depressive and anxiety symptoms during the pandemic while accounting for pre-pandemic symptom burden.

## 2 Materials and methods

### 2.1 Study design and sample

This analysis is based on the Gutenberg COVID-19 Study (GCS), a population-representative, prospective cohort study derived from the Gutenberg Health Study (30).

The GHS is a large-scale, prospective, and representative population study conducted since 2007. The aim of the study is to identify risk factors and causes of the most common diseases. With over 15,000 participants from the Rhine-Main region, it is one of the largest regional health studies in the world. For the GCS, all GHS participants were invited to participate after the onset of the COVID-19 pandemic, enabling a systematic examination of pandemic-related epidemiological effects.

Recruitment for the GHS began in 2007 as a random, stratified sample of residents aged 35–74 years. The sample was stratified by sex, age, and place of residence (Mainz/Mainz-Bingen). Individuals who were mentally or physically unable to visit the study center as well as individuals with low proficiency in the German language were excluded from the study. Two measurement points took place: Baseline (2007–2012; T0) and Follow-Up (2012–2017, T1). In the context of the GCS, two visits to the study center took place, during which a computer-assisted personal interview and sequential sampling of biomaterial were performed. Questionnaires were sent prior to the visit to the study site. The first GCS data collection took place from October 2020 to April 2021 (T2) and the second from March to June 2021 (T3). These assessments coincided with the second and third COVID-19 waves in Germany, a period characterized by changing lockdown measures, night-time curfews and the gradual roll-out of the national vaccination campaign. For the present study, we included respondents with available data for both GHS and GCS measurement waves. This left us with a sample of *N* = 6,813 individuals.

### 2.2 Ethics approval

The requirements of Good Clinical Practice (GCP), Good Epidemiological Practice (GEP), and the ethical standards of the Declaration of Helsinki were considered during the study’s design, implementation, and analysis. Written informed consent was obtained from all participants. Furthermore, the Federal Data Protection Act’s requirements were implemented. The Ethics Committee of the Rhineland-Palatinate Medical Association [GHS: ethics committee of the state medical association RLP; ethics committee vote: 837.020.07(5555)], as well as the Data Protection Officer of the Johannes Gutenberg University Hospital Mainz assessed all study-relevant documentation for the Gutenberg Health Study and the Gutenberg COVID-19 Study and gave a positive vote. The data protection commissioner of Rhineland-Palatinate approved the drawing of the sample via the citizens’ registration offices.

### 2.3 Measurements

#### 2.3.1 Independent variables

Sociodemographic and work-related variables were assessed using computer-assisted personal interviews (CAPI). The sociodemographic variables included sex, age, relationship status, living with minors under 18 years in a household and school degree.

Work-related variables comprised several dimensions: vocational qualification, employment status (e.g., employed, unemployed, retired), volume of employment (e.g., full-time, part-time, no employment), occupational groups. Occupational groups were classified as higher position (academics, employees or civil servants in higher service or leadership positions), lower position (workers, employees or civil servants in basic service), and self-employed. Furthermore, participants were asked about home office arrangements since the beginning of the pandemic, categorized as exclusively working from home, some home office but not all the time, exclusively working on-site at the company.

In addition, changes in work since the pandemic were assessed, including an increase or reduction in weekly working hours, starting a new job, receiving notice of termination, home office either full-time or some days, receiving short-time work compensation, receiving government funds, business closure in the case of self-employment, or a change in income (no change, increased income, reduced income, percentage of change).

#### 2.3.2 Mental health outcomes

Depressiveness was assessed using the Patient Health Questionnaire Depression Scale (31). The questionnaire consists of nine items with a four-point answer scale (0 = “not at all” to 3 = “nearly every day”). The questions refer to the condition of the people within the last two weeks. For evaluation, a sum score and a clinically relevant cut-off (≥10) were calculated.

Anxiety was assessed using the Generalized Anxiety Disorder Scale-2 (32). The questionnaire is a screening instrument consisting of two items with a four-point answer scale (0 = “not at all” to 3 = “nearly every day”). The questions refer to the condition of the people within the last two weeks. For evaluation, a sum score and a clinically relevant cut-off (≥3) were calculated. The PHQ-9 and GAD-2 are commonly applied in epidemiological studies because they demonstrate high validity and reliability in detecting these mental health conditions (33, 34).

### 2.4 Statistical procedure

Descriptive data (absolute and relative frequencies, as well as means with standard deviations) were reported for all variables at the latest available time point (T2 or T3). In order to identify groups particularly affected by the pandemic, we conducted several analyses of covariance (ANCOVA) using the mean value of PHQ-9 and GAD-2 at T2 and T3 as the outcome and the PHQ-9 and GAD-2 at T1 as the covariate in order to control for the pre-pandemic condition toward depressiveness and anxiety. Independent variables were sex (T2), age group (T3), relationship status (T3), minors in the household (yes) (T2), vocational qualification (T2), employment status (T2), volume of employment (T3), occupational group (T2), work-related changes since pandemic (T2), change in net income (T3) and working in home office since the pandemic (T3).

To explore predictors for participants’ distress during the COVID-19 pandemic, two logistic regression analyses (Enter Method) were calculated with the mean dichotomized scores of PHQ-9 and GAD-2 at T2 and T3 as the dependent variables. Independent variables were sex (T2), age (T3), relationship status (T3), occupational group (T2), work-related changes since pandemic (increase and reduction in weekly working hours) (T2), change in net income (T3) and working in home office since the pandemic (T3). Independent variables were dummy coded where needed. Given the exploratory nature of the analyses and the focus on effect sizes and consistent patterns across models, no formal correction for multiple testing was applied. In addition, the PHQ-9 score, respectively, the GAD-2 score for T1 was included as an independent variable in the regression. The logistic regression analyses were restricted to participants who were currently employed. The assumptions for ANCOVA and regression analyses, including normality, homoscedasticity, and absence of multicollinearity, were satisfactorily met. All statistical analyses were conducted using IBM SPSS Statistics 27.

## 3 Results

Descriptive results of the sample are presented in Table 1. *N* = 6,813 persons participated in the study, consisting of 51.2% male and 48.8% female participants with a mean age of 63.5 years (SD = 10.1). The majority (~82%) lived in a partnership, about half of the persons were employed. Among men, 48.2% worked full-time compared to 25.8% of women, while part-time employment was more common among women (26.3% vs. 5.4%). Further sociodemographic and vocational characteristics, including pandemic-related work changes, are detailed in Table 1.

**Table 1:** Sample characteristics at T2/T3.

		
Sex (*N* = 6,813)	*N*	%
Male	3,486	51.2
Female	3,327	48.8
Age in years (*N* = 6,366)	M	SD
Average age	63.5	10.1
Range (minimum/maximum)	45/88	
Age groups (*N* = 6,366)	*N*	%
40–49	491	7.7
50–59	1,994	31.3
60–69	1,991	31.3
70–79	1,450	22.8
≥80	440	6.9
Relationship status (*N* = 5,068)	*N*	%
No	935	18.4
Yes – with joint household	3,745	73.9
Yes – with separated household	388	7.7
Minors in the household (*N* = 3,819)	*N*	%
No	2,873	75.2
Yes	946	24.8
School degree (*N* = 6,810)	*N*	%
General school	1,739	26.1
Secondary school	1,739	25.5
Specific grammar school	769	11.3
Grammar school	2,506	36.8
No school degree	16	0.2
Other degree	2	0.0
Vocational qualification (*N* = 6,810)	*N*	%
Vocational school	2,754	40.4
University	1,678	24.6
College, technical school, master school	1,148	16.9
University of applied sciences	963	14.1
No vocational qualification	248	3.6
Other vocational qualifications	19	0.3
Employment status (*N* = 5,693)	*N*	%
Employed	2,903	42.6
Retired	2,205	33.8
Other employment status	496	7.3
Unemployed	78	1.1
In training	11	0.2
Volume of employment (*N* = 6,094)	*N*	%
Full-time	2,032	33.3
Part-time	837	13.7
Marginally busy	297	4.9
No employment	2,928	48.0
Occupational groups* (*N* = 3,339)	*N*	%
Higher position	1,502	45.0
Lower position	1,411	42.2
self-employed	426	12.8
Home office (*N* = 3005)	*N*	%
Only HO	615	20.5
Some HO but not all the time	952	31.7
Only in the company	1,438	47.9
Work-related changes since pandemic (T2) (*N* = 3,858)	*N*	%
Increase in weekly working hours	413	10.7
Reduction in weekly working hours	387	10.1
New job	70	1.8
Notice of termination	37	1.0
Home office all or some days	1,457	37.4
Short-time work compensation	350	9.2
Government funds	96	2.6
For self-employed: business closes	76	2.0
For self-employed: reduction in income	298	7.6
Equivalized income (per person & month)	*N*	M (SD) [€]
	6,113	3009 (2020)
Change in net income (*N* = 5,698)	*N*	%
No change in net income	4,996	87.7
Increased net income	244	4.3
Reduced net income	458	8.0

Sample sizes vary across variables because some items were assessed only among currently employed participants. *Occupational groups: higher position: academics, employees/civil servants higher service, leading position; lower position: workers, employees/civil servants’ in basic service.

### 3.1 PHQ-9/GAD-2 before and during the COVID-19 pandemic

Table 2 summarizes depressive (PHQ-9) and anxiety (GAD-2) symptoms at four time points (twice before and twice during the pandemic).

**Table 2:** PHQ-9/GAD-2 scores at the timepoints from T0 to T3.

Outcome* PHQ-9	T0 GHS BL (*N* = 6,554)	T1 GHS FU (*N* = 6,446)	T2 GCS BL (*N* = 6,114)	T3 GCS FU (*N* = 5,920)
PHQ-9 (M/SD)	3.92 (3.41)	4.22 (3.51)	4.06 (3.65)	4.14 (3.87)
PHQ-9 ≥ 5 (%/*N*)	32.5% (2131)	37.4% (2412)	35.6% (2175)	36.3% (2149)
PHQ-9 ≥ 10 (%/*N*)	7.0% (457)	7.6% (491)	8.1% (491)	9.2% (544)
PHQ-9 ≥ 15 (%/*N*)	1.5% (100)	1.9% (124)	2.0% (120)	2.5% (148)
PHQ-9 ≥ 20 (%/*N*)	0.4% (24)	0.4% (23)	0.5% (29)	0.5% (31)

Outcome GAD-2	T0 GHS BL (*N* = 6,751)	T1 GHS FU (*N* = 6,756)	T2 GCS BL *(N = 4,650)*	T3 GCS FU *(N = 6,122)*
GAD-2 (M/SD)	0.85 (1.09)	0.95 (1.08)	0.77 (1.09)	0.73 (1.08)
GAD-2 ≥ 3 (%/*N*)	6.3% (422)	6.5% (441)	6.0% (277)	5.5% (336)

*PHQ-9 (Patient Health Questionnaire, depressive symptoms), GAD-2 (General anxiety disorder, anxiety symptoms), T0: 10.04.2007–13.04.2012, T1: 27.04.2012–28.04.2017, T2: 12.10.2020–09.04.2021, T3: 01.03.2021–26.06.2021.

Mean PHQ-9 and GAD-2 scores changed only marginally across assessments. However, the proportion of participants with clinically relevant depressive symptoms (PHQ-9 ≥ 10) increased during the pandemic (T2, T3) compared to pre-pandemic measurements (T0, T1). No such increase was observed for GAD-2 (≥3) cases (see Table 2).

**Figure 1:**
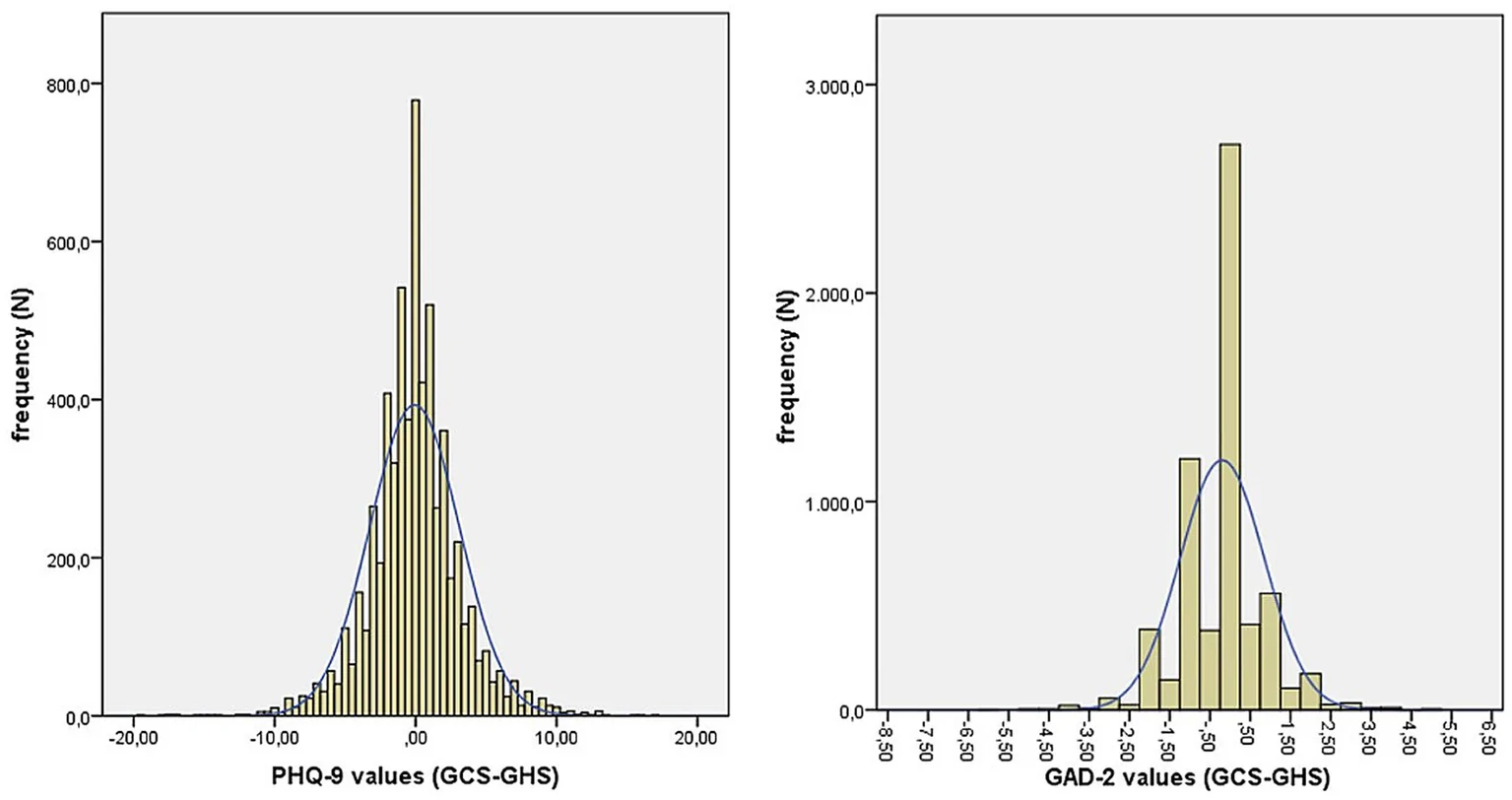
Intra-individual differences between PHQ-9 values (left) and GAD-2 values (right) before and during the pandemic. Values were calculated with individual GCS mean values (T2/T3) minus individual GHS values (T1) (*x*-axis), *y*-axis: *N*; mean differences PHQ-9: *M* = −0.07; *SD* = 3.18; *N* = 6271; mean differences GAD-2: *M* = −0.19; *SD* = 1.05; *N* = 6530.

Figure 1 illustrates intra-individual changes in PHQ-9 and GAD-2 scores comparing before and during the pandemic. Most participants showed no or minimal changes in depressive symptoms, with roughly equal proportions experiencing improvement or deterioration. A similar pattern was observed for anxiety symptoms; only a slight shift toward more participants improving was detected.

### 3.2 (Vocational) predictors of participants’ distress during the COVID-19 pandemic

One central aim of our study was to identify (vocational) predictors of participants’ distress. Table 3 shows the results of the ANCOVAS that had small-to-negligible effect sizes. All other ANCOVAs with significant independent variables but very small effect sizes are displayed in Supplementary Table 1. Sex and age group showed significant differences in PHQ-9 and GAD-2 when controlling for pre-pandemic distress. Considering the PHQ-9 and GAD-2 values before the pandemic, being female and middle-aged was associated with higher distress. The mean value for depressive symptoms was comparable before vs. during the pandemic in women, while men showed very slightly lower values during the pandemic. The mean values for anxiety symptoms were slightly lower during the pandemic compared to before in men and women (see Table 3). Furthermore, participants with an increase in weekly working hours since the pandemic had significant higher scores in PHQ-9 and GAD-2. The volume of employment showed significant associations with depressive and anxiety symptoms as well: post-hoc tests revealed working part-time compared to all other categories went along with higher depressive and anxiety symptoms. Additionally, working full-time versus being unemployed was associated with higher depressive but not anxiety symptoms. With regards to depressive symptoms, further differences were observed in terms of employment status, relationship status, and occupational group. Participants who were employed showed significantly more depressive symptoms than those who were retired. A lower depressive score also went along with living in joint households. Self-employed individuals exhibited significantly fewer symptoms than individuals in lower positions.

**Table 3:** Overview of the key figures for all ANCOVAs performed to identify groups particularly affected by the pandemic with at least a small effect size.

PHQ-9	GHS FU M (SD) [T1]	GCS M (SD) [T2/3]	F (df)	*p*	*Cohen’s d*	Explanations/Post-hoc
Sex	m = 3.76 (3.40) *f* = 4.72 (3.55)	m = 3.55 (3.41) *f* = 4.73 (3.70)	66.97 (1;6,268)	***	0.21	-
Age group	40–49 = 4.23 (3.54) 50–59 = 4.49 (3.64) 60–69 = 4.46 (3.59) 70–79 = 3.53 (3.05) ≥80 = 3.48 (2.77)	40–49 = 4.60 (3.84) 50–59 = 4.65 (3.80) 60–69 = 3.91 (3.45) 70–79 = 3.46 (3.27) ≥80 = 3.60 (3.23)	20.34 (4;5,932)	***	0.24	40–49 *60–69/70–79 (<0.001, <0.001) 50–59 * 60–69/70–79 (<0.001)
Increase in weekly working hours	Yes = 4.59 (3.61) No = 4.35 (3.60)	Yes = 5.35 (3.86) No = 4.25 (3.65)	38.48 (1; 3,678)	***	0.20	-
Volume of employment	Full-time = 4.16 (3.49) Part-time = 4.62 (3.42) Marginally busy = 4.43 (3.51) Unemployed = 4.02 (3.42)	Full-time = 4.17 (3.59) Part-time = 4.93 (3.57) Marginally busy = 4.27 (3.65) Unemployed = 3.76 (3.51)	17.09 (3; 5,717)	***	0.19	Full-time vs. unemployed (<0.001) part-time * full-time /marginally busy/no employment (<0.001, 0.03, <0.001)
Employment status	Employed = 4.36 (3.54) Unemployed = 5.56 (3.85) In training = 4.55 (4.87) Retired = 3.92 (3.39) other = 4.57 (3.51)	Employed = 4.43 (3.62) Unemployed = 5.47 (4.16) In training = 4.00 (4.27) Retired = 3.65 (3.36) other = 4.27 (3.93)	10.74 (4; 5,413)	***	0.18	Employed * retired (<0.001)
Relationship status	No relationship = 4.83 (3.89) Yes, joint household = 4.06 (3.37) Yes, separated = 4.90 (4.02)	No relationship = 4.84 (4.06) Yes, Joint household = 3.94 (3.38) Yes, separated = 5.19 (4.09)	16.47 (2; 4,739)	***	0.17	Yes, joint household* yes, separated households/no relationship (<0.001, <0.001)
Occupational groups	Self-employed = 3.93 (3.50) higher position = 3.98 (3.27) lower position = 4.77 (3.74)	Self-employed = 3.80 (3.74) higher position = 4.09 (3.40) lower position = 4.78 (3.88)	6.01 (2; 3,164)	***	0.16	Self-employed *lower position (0.04)

GAD-2	GHS FU M (SD) [T1]	GCS M (SD) [T2/3]	*F* (df)	*p*	*Cohen’s d*	Post-hoc
Sex	m = 0.79 (1.02) *f* = 1.11 (1.13)	m = 0.59 (0.91) *f* = 0.92 (1.11)	70.30 (1;6,527)	***	0.21	-
Volume of employment	Full-time = 0.96 (1.08) part-time = 1.09 (1.15) marginally busy = 0.94 (1.11) unemployed = 0.89 (1.05)	Full-time = 0.72 (1.00) part-time = 1.00 (1.18) marginally busy = 0.77 (1.00) unemployed = 0.68 (0.97)	15.33 (3;6,011)	***	0.18	part-time * full-time/marginally busy/no employment (<0.001, 0.02; <0.001)
Increase in weekly working hours	Yes = 1.08 (1.25) No = 0.98 (1.10)	Yes = 1.06 (1.19) No = 0.77 (1.05)	27.02 (1;3,767)	***	0.17	-
Age group	40–49 = 0.95 (1.09) 50–59 = 1.01 (1.12) 60–69 = 1.03 (1.08) 70–79 = 0.76 (0.98) ≥80 = 0.70 (0.88)	40–49 = 0.89 (1.14) 50–59 = 0.84 (1.09) 60–69 = 0.72 (1.01) 70–79 = 0.61 (0.90) ≥80 = 0.61 (0.83)	9.41 (4;6,212)	***	0.16	40–49 * 60–69/70–79/≥80 (<0.001, <0.001, 0.04) 50–59 * 60–69/70–79 (<0.001, 0.006)

*Indicate post-hoc compared categories; underlined variables show lower PHQ-9/GAD-2 values; GCS [T2/3]: mean value of T2 & T3; *p < 0.05, **p < 0.01, ***p < 0.001. Cohen’s d values were derived from partial eta squared and should therefore be interpreted as approximate d-equivalent effect sizes, particularly for predictors with more than two categories.

Separate ANCOVA analyses for T2 and T3 yielded highly similar patterns of associations compared with the analyses based on averaged T2/T3 scores. Effect sizes differed only marginally across the two pandemic assessment periods and remained within the same small-to-negligible range for all predictors (Supplementary Table 2).

Two logistic regression models were computed to identify predictors for depression (PHQ-9 ≥ 10) and anxiety (GAD-2 ≥ 3). The variance explained by the model (Nagelkerke R^2^) for the two analyses was 0.28 and 0.23, respectively. Among employed participants, pre-pandemic depressive symptoms (OR = 1.33), an increase in weekly working hours (OR = 2.49) and partnership status (being without a partner; OR = 2.12) significantly predicted having clinically relevant symptoms of depression during the pandemic (see Table 4). Among employed participants, higher pre-pandemic anxiety symptoms (OR = 2.24) and an increase in weekly working hours (OR = 1.86) significantly predicted clinically relevant anxiety symptoms during the pandemic (Table 4). The T1 scores of PHQ-9 and GAD-2, respectively, were by far the strongest predictors in both regressions. When only PHQ-9, respectively, GAD-2 at T1 was included as an independent variable, the model explained 28%, respectively, 22% of the variance.

**Table 4:** Significant predictors for PHQ-9 and GAD-2 above the cutoff (logistic regression model).

PHQ-9 ≥ 10 (R^2^ = 0.28)	OR (95% CI)	*p*
PHQ-9 (pre-pandemic)	1.33 (1.27–1.40)	< 0.001
Increase in weekly working hours	2.49 (1.56–4.00)	< 0.001
Relationship status [alone]	2.12 (1.33–3.38)	0.001

GAD-2 ≥ 3 (R^2^ = 0.23)	OR (95% CI)	*p*
GAD-2 (pre-pandemic)	2.24 (1.92–2.63)	< 0.001
Increase in weekly working hours	1.86 (1.07–3.24)	0.028

The effective sample sizes were *N* = 1,688 for the PHQ-9 model and *N* = 1,709 for the GAD-2 model.

## 4 Discussion

This study examined depressive and anxiety symptoms before and during the COVID-19 pandemic in a large population-based cohort of middle-aged and older adults. By linking pre-pandemic mental health assessments with data collected during the second and third COVID-19 waves in Germany, we investigated whether vocational and sociodemographic factors were associated with mental health during the pandemic after accounting for pre-pandemic symptom burden. The pandemic assessments at T2 and T3 covered roughly the first half of the pandemic, during which public health restrictions, infection dynamics, and vaccination coverage were changing rapidly.

Mean depressive and anxiety symptom scores changed only slightly from the pre-pandemic to the pandemic assessments. However, the proportion of participants with clinically relevant depressive symptoms increased during the pandemic, whereas no comparable increase was observed for clinically relevant anxiety symptoms. At the individual level, most participants showed no or only minimal change, while some experienced deterioration and others showed improvement. This pattern is broadly consistent with longitudinal research in middle-aged and older adults showing that depressive symptoms may persist longer, whereas anxiety symptoms may recover more rapidly over the course of the pandemic (24, 25).

Previous studies showed a decline of mental health in the early stages of the pandemic in the general population, followed by an improvement in the following months (26, 27, 35). In this context, the absence of pronounced average changes is an important finding, particularly in light of early cross-sectional studies suggesting more substantial and uniform pandemic-related deterioration in mental health. Our findings are consistent with longitudinal evidence indicating smaller and more heterogeneous changes than initially suggested by cross-sectional studies (26, 35, 36). They are also consistent with a systematic review and meta-analysis in adults aged 60 years and older, which found only small average increases in mental distress during the early pandemic and evidence of subsequent adaptation or recovery processes (25). Similarly, a study observed distinct trajectories of depressive and anxiety symptoms in middle-aged and older adults, with depressive symptoms persisting more strongly over time while anxiety symptoms improved (24).

Separate analyses for T2 and T3 yielded highly similar patterns of associations compared with the main analyses using the averaged T2/T3 outcomes. Effect sizes differed only marginally between the two pandemic assessment periods and remained within the same small-to-negligible range across all predictors. The similarity of the findings should also be considered in light of the relatively close and partially overlapping assessment windows of T2 and T3, which may have limited the extent to which distinct phase-specific differences emerged. Thus, although averaging T2 and T3 may reduce information on short-term fluctuations, the separate analyses indicate that the central findings were not driven by either pandemic assessment period alone.

Pre-pandemic depressive and anxiety symptoms were strongly associated with the corresponding clinically relevant symptoms during the pandemic. This longitudinal continuity is notable given the long interval between the pre-pandemic and pandemic assessments and the substantial societal disruptions caused by COVID-19. In the regression models restricted to employed participants, pre-pandemic depressive and anxiety scores remained the strongest predictors of the corresponding pandemic-period outcomes. The logistic regression models explained 28 and 22% of the variance in clinically relevant depression and anxiety, respectively, with pre-pandemic PHQ-9 and GAD-2 scores emerging as the strongest predictors. Importantly, the PHQ-9 and GAD-2 assess symptom severity during the previous two weeks and are widely used not only for screening purposes but also for monitoring changes in symptom severity over time. The observed associations should therefore not be interpreted as a mere methodological consequence of the instruments. This finding is consistent with longitudinal research showing that individuals with pre-pandemic depressive symptoms were at increased risk of persistent or worsening mental health problems during the pandemic (37). At the same time, the substantial heterogeneity across individual trajectories indicates that pre-pandemic symptom burden should be understood as an important vulnerability marker rather than as determining an inevitable deterioration in mental health.

### 4.1 Vocational predictors of distress

An increase in weekly working hours was consistently associated with higher depressive and anxiety scores during the pandemic after accounting for pre-pandemic symptom burden. The association was also observed in the regression models restricted to employed participants, although the work-related predictors explained comparatively little additional variation beyond pre-pandemic mental health. This finding is consistent with previous research linking increased working hours and higher work intensity during the pandemic to greater psychological distress, although much of this evidence was derived from healthcare and other particularly exposed occupational groups (10–15). Increased working hours may have reduced opportunities for rest and recovery or reflected higher occupational demands and additional role conflicts. In addition, increased working hours may have occurred more frequently in occupations that were particularly affected by the pandemic and associated with higher workload, greater responsibility, or more direct exposure to its consequences, as reported in previous research (38). However, the present data do not provide information on the reasons for changes in working hours, whether these changes were voluntary, or which specific occupational demands were involved. These explanations therefore remain tentative. At the same time, the association between increased working hours and distress contrasts with other previous findings showing poorer mental health among workers aged 55 years and older who experienced reductions in working hours or income (28). These contrasting findings may reflect differences in study populations, labor-market conditions, pandemic phases, or the underlying circumstances of working-time changes. Reductions in working hours may indicate job insecurity and financial strain, whereas increases in working hours may reflect greater workload and occupational demands. Taken together, these findings suggest that changes in working hours in either direction may be relevant for mental health, but that their meaning depends on the broader employment context. However, although increased working hours emerged as the most consistent work-related correlate of distress across analyses, the observed effect sizes remained small, suggesting that this played only a limited role relative to other individual and contextual factors.

Home office arrangements showed only a negligible association with depressive symptoms and did not emerge as an independent predictor in the regression models. This finding should be interpreted cautiously, as the study did not assess the quality or individual circumstances of working from home. A systematic review similarly found heterogeneous physical and psychological effects of remote working during the COVID-19 pandemic and emphasized the generally limited certainty of the available evidence (16, 17, 39).

The ANCOVA results further indicated differences according to employment status and volume of employment. Participants working part-time showed higher depressive and anxiety symptom levels than all other employment-volume groups. In addition, participants working full-time showed higher depressive symptom levels than those with no employment, while employed participants showed higher depressive symptom levels than retired participants. Another study also found a relative increase in mental distress in persons who were employed before the pandemic compared to those who were unemployed (40). These findings should be interpreted in light of the age structure of the cohort. Among middle-aged and older adults, those who were unemployed or retired may have been less directly exposed to abrupt pandemic-related changes in working conditions, whereas employed participants had to adapt to changes in workload, job insecurity, work organization, infection risk, or the transition to remote work. Persons who were unemployed or retired before the pandemic may have maintained a more stable daily routine, rendering them less affected by such disruptions. Part-time employment was more common among women in our sample and may also have been associated with caregiving and domestic responsibilities. The higher symptom burden observed among part-time workers could therefore partly reflect the unequal distribution of unpaid care work rather than the effect of part-time employment itself. Working part-time often comes along with more domestic work or caregiving responsibilities, which results in double load (41). Because sex-stratified and interaction analyses were beyond the scope of the present study, this interpretation remains tentative.

The only significant difference between occupational groups was that self-employed participants reported fewer depressive symptoms than participants in lower occupational positions. Individuals in lower occupational positions may have had less autonomy and control over their working environment, fewer opportunities to work from home, and greater occupational exposure during the pandemic. They may also have been more vulnerable to economic strain during the pandemic (9). However, these potential mechanisms were not directly assessed and should therefore be interpreted cautiously.

Income reductions were associated with slightly higher depressive and anxiety symptom levels in the ANCOVA analyses, but the corresponding effect sizes were very small. Moreover, reduced net income was no longer a significant predictor of clinically relevant anxiety symptoms in the regression model restricted to employed participants. This suggests that income changes made only a limited independent contribution after accounting for pre-pandemic symptom burden and the other variables included in the model. The comparatively high educational level and average income of the cohort may have buffered some of the adverse effects of financial strain and may limit the generalizability of this finding to more socioeconomically disadvantaged populations.

Overall, vocational factors showed small to negligible associations with mental health in this middle-aged and older cohort. The strongest and most consistent work-related association concerned increased weekly working hours, whereas associations with employment volume, occupational position, and income changes were comparatively small and partly dependent on the analytical approach. Taken together, these findings indicate that the vocational factors examined in this study played only a limited role in pandemic-period mental health after pre-pandemic symptom burden had been taken into account.

### 4.2 Sociodemographic predictors of distress

Women showed higher depressive and anxiety symptoms during the pandemic after accounting for pre-pandemic symptom burden. This finding is consistent with previous research identifying women as a particularly vulnerable subgroup during the pandemic (40, 42–46). Importantly, this pattern has also been observed specifically in middle-aged and older populations. Longitudinal studies and a systematic review of adults aged 60 years and older found greater increases in mental distress among women than among men (22, 24, 25). Potential explanations include women’s greater representation in economically affected sectors and healthcare occupations, as well as their disproportionate share of caregiving and domestic responsibilities (1, 47, 48), which was already the case before the pandemic, but has been dramatically increased during the pandemic (48–50). However, because work and caregiving demands were not examined jointly in the present analyses, the mechanisms underlying the observed sex differences cannot be determined from our data.

Within our middle-aged and older cohort, participants in the middle-aged groups showed higher depressive and anxiety symptom levels than older participants. This finding is consistent with evidence suggesting that older adults often experienced smaller average changes in mental distress and showed indications of adaptation or recovery during the pandemic (24, 25). In contrast, middle-aged adults may have been more directly exposed to the simultaneous demands of employment, financial responsibilities, and caregiving for children or older family members. Research conducted during the pandemic identified parenting and unpaid caregiving responsibilities as relevant correlates of psychological distress (20, 21, 23). Nevertheless, these explanations remain tentative because the present study did not assess the combined contribution of employment, financial responsibilities, and caregiving demands.

Relationship and household circumstances were also associated with mental health. Participants who did not live with a partner showed higher depressive and anxiety symptom levels, and living alone was associated with a higher likelihood of clinically relevant depressive symptoms. These findings are consistent with longitudinal studies identifying individuals living alone as a vulnerable subgroup among middle-aged and older adults during the pandemic (22, 24). This aligns with findings that older adults in relationships or with children exhibited higher psychological resilience during the pandemic (51). Contact restrictions during the pandemic likely increased social isolation and reduced social support opportunities for those living alone or in separate households compared to shared households. This may have led to increases in distress as it is strongly linked to loneliness (52–54).

Overall, the findings indicate that sociodemographic vulnerability within middle-aged and older populations was not uniform: women, middle-aged participants, and individuals without a partner or living alone showed comparatively higher symptom levels, whereas older age appeared to be associated with lower average distress in this cohort.

### 4.3 Strengths and limitations

A major strength of this study is the availability of repeated assessments of depressive and anxiety symptoms before and during the COVID-19 pandemic within the same participants. The large population-based cohort of more than 6,000 middle-aged and older adults also provided detailed sociodemographic and vocational information, allowing pandemic-period mental health to be examined while accounting for pre-pandemic symptom burden. Also, our results complement other studies in which older people were underrepresented (37).

Several limitations should be considered. First, T2 and T3 covered relatively long, temporally close, and partially overlapping assessment periods during a dynamic phase of the pandemic. We averaged both assessments in the main analyses to obtain a stable estimate of mental health during this period, which may have obscured short-term fluctuations. However, separate T2 and T3 analyses yielded highly similar directions and effect sizes, indicating that the central findings were not driven by either assessment period alone (Supplementary Table 2).

Second, T2 and T3 were conducted during the second and third COVID-19 waves in Germany. The findings may therefore not be generalized to later pandemic or post-pandemic periods. Moreover, the cohort consisted of middle-aged and older adults and included comparatively well-educated and higher-income participants. The results should consequently not be generalized to younger employees or populations with more precarious employment and fewer socioeconomic resources. The workforce regression analyses were additionally restricted to employed participants with complete data and therefore apply specifically to this subsample.

Finally, although we included a broad range of vocational factors, potentially relevant working conditions such as job autonomy, workplace social support, leadership quality, perceived workplace safety, and the reasons for changes in working hours were not assessed. All measures were based on self-report and did not include clinical diagnoses or objective working conditions. Although the PHQ-9 and GAD-2 are valid instruments for assessing and monitoring symptoms, the limited range and low mean scores of the GAD-2 may have contributed to floor effects and reduced sensitivity to small changes in anxiety symptoms.

## 5 Conclusion

In this large population-based cohort of middle-aged and older adults, mean depressive and anxiety symptom levels changed only slightly from before to during the COVID-19 pandemic, although the proportion of participants with clinically relevant depressive symptoms increased modestly. Individual trajectories were heterogeneous: most participants showed no or only minimal change from their pre-pandemic symptom levels, while some experienced deterioration and others showed improvement. These findings indicate that relatively stable average symptom levels can coexist with clinically relevant changes in vulnerable subgroups. Prevention should therefore not be guided solely by average population-level changes but should also address subgroups and individuals who show deterioration or require additional support.

Pre-pandemic depressive and anxiety symptoms were strongly associated with the corresponding outcomes during the pandemic. In contrast, the vocational factors examined in this study showed only small to negligible additional associations after pre-pandemic symptom burden had been taken into account. Increased weekly working hours emerged as the most consistent work-related correlate of depressive and anxiety symptoms among employed participants. The workplace may nevertheless provide an accessible setting for mental health promotion among employed adults, for example by raising awareness of psychological strain, facilitating access to support, and monitoring excessive or changing workloads. Given the limited effect sizes observed for vocational factors, workplace-based measures should form part of a broader preventive approach rather than being regarded as the primary response to pandemic-related mental health burden.

Women, middle-aged participants within the cohort, and individuals without a partner or living alone showed comparatively higher symptom levels and may represent groups requiring particular attention during public health crises. Preventive approaches should therefore consider both prior mental health and social circumstances rather than focusing on occupational factors alone.

Future research should examine whether vocational factors play a greater role in younger working-age populations and in groups with more precarious employment, lower income, or fewer socioeconomic resources. More differentiated analyses, including interaction and subgroup analyses by sex, occupational group, caregiving responsibilities, and stage of working life, may help identify whether associations between vocational circumstances and mental health vary across population groups and under which conditions these associations become particularly relevant.

## Data Availability

The datasets generated and/or analyzed during the current study are not publicly available due to the restrictions documented in the written consent form of the GCS study participants but are available from the corresponding author on reasonable request.

## References

[1] International Labour Organization. (2020) ILO Monitor: COVID-19 and the world of work. Fourth edition: Updated estimates and analysis. Available online at: https://www.ilo.org/sites/default/files/wcmsp5/groups/public/%40dgreports/%40dcomm/documents/briefingnote/wcms:745963.pdf (Accessed August 19, 2026).

[2] Wang S Kamerāde D Bessa I Burchell B Gifford J Green M . The impact of reduced working hours and furlough policies on workers’ mental health at the onset of COVID-19 pandemic: a longitudinal study. J Soc Pol. (2024) 53:702–26. doi: 10.1017/S0047279422000599

[3] Ringlein GV Ettman CK Stuart EA. Income or job loss and psychological distress during the COVID-19 pandemic. JAMA Netw Open. (2024) 7:e2424601. doi: 10.1001/jamanetworkopen.2024.24601, 39078628 PMC11289696

[4] Lai S Lu L Shen C Yan A Lei Y Zhou Z . Income loss and subsequent poor psychological well-being among the Chinese population during the early COVID-19 pandemic. Int J Equity Health. (2023) 22:219. doi: 10.1186/s12939-023-02022-1, 37848883 PMC10583462

[5] Beese F Wachtler B Grabka MM Blume M Kersjes C Gutu R . Socioeconomic inequalities in pandemic-induced psychosocial stress in different life domains among the working-age population. BMC Public Health. (2024) 24:1421. doi: 10.1186/s12889-024-18874-3, 38807100 PMC11131271

[6] Ettman CK Abdalla SM Cohen GH Sampson L Vivier PM Galea S. Prevalence of depression symptoms in US adults before and during the COVID-19 pandemic. JAMA Netw Open. (2020) 3:e2019686. doi: 10.1001/jamanetworkopen.2020.19686, 32876685 PMC7489837

[7] Riehm KE Holingue C Smail EJ Kapteyn A Bennett D Thrul J . Trajectories of mental distress among U.S. adults during the COVID-19 pandemic. Ann Behav Med. (2021) 55:93–102. doi: 10.1093/abm/kaaa126, 33555336 PMC7929474

[8] Wilson JM Lee J Fitzgerald HN Oosterhoff B Sevi B Shook NJ. Job insecurity and financial concern during the COVID-19 pandemic are associated with worse mental health. J Occup Environ Med. (2020) 62:686–91. doi: 10.1097/jom.0000000000001962, 32890205

[9] Petersen J Hettich N Baumkötter R Wild PS Pfeiffer N Münzel T . The burdens of poverty during the COVID-19 pandemic. Front Sociol. (2022) 7:995318. doi: 10.3389/fsoc.2022.995318, 36505762 PMC9731111

[10] Vizheh M Qorbani M Arzaghi SM Muhidin S Javanmard Z Esmaeili M. The mental health of healthcare workers in the COVID-19 pandemic: a systematic review. J Diabetes Metab Disord. (2020) 19:1967–78. doi: 10.1007/s40200-020-00643-9, 33134211 PMC7586202

[11] Spoorthy MS Pratapa SK Mahant S. Mental health problems faced by healthcare workers due to the COVID-19 pandemic-a review. Asian J Psychiatr. (2020) 51:102119. doi: 10.1016/j.ajp.2020.102119, 32339895 PMC7175897

[12] Du J Mayer G Hummel S Oetjen N Gronewold N Zafar A . Mental health burden in different professions during the final stage of the COVID-19 lockdown in China: cross-sectional survey study. J Med Internet Res. (2020) 22:e24240. doi: 10.2196/24240, 33197231 PMC7713530

[13] Lavell AHA Sikkens JJ Buis DT Smulders YM Vinkers CH Bomers MK . Mental health of health care workers during and after the COVID-19 pandemic – a longitudinal cohort study. PLoS Ment Health. (2025) 2:e0000333. doi: 10.1371/journal.pmen.0000333, 41661990 PMC12798477

[14] Mohr DC Elnahal S Marks ML Derickson R Osatuke K. Burnout trends among US health care workers. JAMA Netw Open. (2025) 8:e255954. doi: 10.1001/jamanetworkopen.2025.5954, 40257797 PMC12013355

[15] Caramello V Gariglio V Di Salvo G Maina G Boccuzzi A. Longitudinal assessment of mental health consequences of the COVID-19 pandemic long-term exposure on health care workers from a north west Italian hospital. Disaster Med Public Health Prep. (2023) 17:e378. doi: 10.1017/dmp.2023.42, 36891915 PMC10125871

[16] Eurofound and European Commission Joint Research Centre. What Just Happened? COVID-19 Lockdowns and Change in the Labour Market. Luxembourg: Eurofound and European Commission Joint Research Centre (2021).

[17] Zhang C Yu MC Marin S. Exploring public sentiment on enforced remote work during COVID-19. J Appl Psychol. (2021) 106:797–810. doi: 10.1037/apl0000933, 34138587

[18] Singhal S Kumar P Singh S Saha S Dey AB. Clinical features and outcomes of COVID-19 in older adults: a systematic review and meta-analysis. BMC Geriatr. (2021) 21:321. doi: 10.1186/s12877-021-02261-3, 34011269 PMC8133052

[19] Vahia IV Jeste DV Reynolds CF. Older adults and the mental health effects of COVID-19. JAMA. (2020) 324:2253–4. doi: 10.1001/jama.2020.21753, 33216114

[20] Gadermann AC Thomson KC Richardson CG Gagné M McAuliffe C Hirani S . Examining the impacts of the COVID-19 pandemic on family mental health in Canada: findings from a national cross-sectional study. BMJ Open. (2021) 11:e042871. doi: 10.1136/bmjopen-2020-042871, 33436472 PMC7804831

[21] Hettich-Damm N Petersen J Zahn D Baumkoetter R Wild PS Muenzel T . Gender differences and the impact of partnership and children on quality of life during the COVID-19 pandemic. Int J Public Health. (2023) 68:1605826. doi: 10.3389/ijph.2023.1605826, 37284508 PMC10239859

[22] Zaninotto P Iob E Demakakos P Steptoe A. Immediate and longer-term changes in the mental health and well-being of older adults in England during the COVID-19 pandemic. JAMA Psychiatry. (2022) 79:151–9. doi: 10.1001/jamapsychiatry.2021.3749, 34935862 PMC8696687

[23] Czeisler MÉ Rohan EA Melillo S Matjasko JL DePadilla L Patel CG . Mental health among parents of children aged <18 years and unpaid caregivers of adults during the COVID-19 pandemic - United States, December 2020 and February-march 2021. MMWR Morb Mortal Wkly Rep. (2021) 70:879–87. doi: 10.15585/mmwr.mm7024a3, 34138835 PMC8220951

[24] Mooldijk SS Dommershuijsen LJ de Feijter M Luik AI. Trajectories of depression and anxiety during the COVID-19 pandemic in a population-based sample of middle-aged and older adults. J Psychiatr Res. (2022) 149:274–80. doi: 10.1016/j.jpsychires.2022.03.002, 35305381 PMC8906533

[25] Schäfer SK Lindner S Kunzler AM Meerpohl JJ Lieb K. The mental health impact of the COVID-19 pandemic on older adults: a systematic review and meta-analysis. Age Ageing. (2023) 52:afad170. doi: 10.1093/ageing/afad170, 37725975

[26] Robinson E Sutin AR Daly M Jones A. A systematic review and meta-analysis of longitudinal cohort studies comparing mental health before versus during the COVID-19 pandemic in 2020. J Affect Disord. (2022) 296:567–76. doi: 10.1016/j.jad.2021.09.098, 34600966 PMC8578001

[27] Salanti G Peter NL Tonia T Holloway A Darwish L Kessler RC . Changes in the prevalence of mental health problems during the first year of the pandemic: a systematic review and dose-response meta-analysis. BMJ Mental Health. (2024) 27:e301018. doi: 10.1136/bmjment-2024-301018, 38876492 PMC11177678

[28] Abrams LR Finlay JM Kobayashi LC. Job transitions and mental health outcomes among U.S. adults aged 55 and older during the COVID-19 pandemic. J Gerontol B Psychol Sci Soc Sci. (2022) 77:e106–16. doi: 10.1093/geronb/gbab060, 33837416 PMC8083363

[29] Rinsky-Halivni L Brammli-Greenberg S Christiani DC. Ageing workers' mental health during COVID-19: a multilevel observational study on the association with the work environment, perceived workplace safety and individual factors. BMJ Open. (2022) 12:e064590. doi: 10.1136/bmjopen-2022-064590, 36572502 PMC9805828

[30] Wild PS Zeller T Beutel M Blettner M Dugi KA Lackner KJ . Die Gutenberg Gesundheitsstudie. Bundesgesundheitsblatt Gesundheitsforschung Gesundheitsschutz. (2012) 55:824–30. doi: 10.1007/s00103-012-1502-7, 22736163

[31] Löwe B Spitzer RL Zipfel S Herzog W. Gesundheitsfragebogen für Patienten (PHQ-D). Komplettversion und Kurzform [Testmappe mit Manual, Fragebögen, Schablonen]. 2. Auflage ed. Karlsruhe: Pfizer (2002).

[32] Löwe B Wahl I Rose M Spitzer C Glaesmer H Wingenfeld K . A 4-item measure of depression and anxiety: validation and standardization of the patient health Questionnaire-4 (PHQ-4) in the general population. J Affect Disord. (2010) 122:86–95. doi: 10.1016/j.jad.2009.06.019, 19616305

[33] Kliem S Sachser C Lohmann A Baier D Brähler E Gündel H . Psychometric evaluation and community norms of the PHQ-9, based on a representative German sample. Front Psych. (2024) 15:1483782. doi: 10.3389/fpsyt.2024.1483782 PMC11670475 39726913

[34] Plummer F Manea L Trepel D McMillan D. Screening for anxiety disorders with the GAD-7 and GAD-2: a systematic review and diagnostic metaanalysis. Gen Hosp Psychiatry. (2016) 39:24–31. doi: 10.1016/j.genhosppsych.2015.11.005 26719105

[35] Ahmed N Barnett P Greenburgh A Pemovska T Stefanidou T Lyons N . Mental health in Europe during the COVID-19 pandemic: a systematic review. Lancet Psychiatry. (2023) 10:537–56. doi: 10.1016/S2215-0366(23)00113-X, 37321240 PMC10259832

[36] Hettich N Entringer TM Kroeger H Schmidt P Tibubos AN Braehler E . Impact of the COVID-19 pandemic on depression, anxiety, loneliness, and satisfaction in the German general population: a longitudinal analysis. Soc Psychiatry Psychiatr Epidemiol. (2022) 57:2481–90. doi: 10.1007/s00127-022-02311-0, 35680681 PMC9181932

[37] Benke C Asselmann E Entringer TM Pané-Farré CA. The role of pre-pandemic depression for changes in depression, anxiety, and loneliness during the COVID-19 pandemic: results from a longitudinal probability sample of adults from Germany. Eur Psychiatry. (2022) 65:e76. doi: 10.1192/j.eurpsy.2022.2339, 36325825 PMC9706309

[38] Umbetkulova S Kanderzhanova A Foster F Stolyarova V Cobb-Zygadlo D. Mental health changes in healthcare workers during COVID-19 pandemic: a systematic review of longitudinal studies. Eval Health Prof. (2024) 47:11–20. doi: 10.1177/01632787231165076, 37143216 PMC10160822

[39] Wells J Scheibein F Pais L Rebelo Dos Santos N Dalluege C-A Czakert JP . A systematic review of the impact of remote working referenced to the concept of work-life flow on physical and psychological health. Workplace Health Saf. (2023) 71:507–21. doi: 10.1177/21650799231176397, 37387511 PMC10612377

[40] Pierce M Hope H Ford T Hatch S Hotopf M John A . Mental health before and during the COVID-19 pandemic: a longitudinal probability sample survey of the UK population. Lancet Psychiatry. (2020) 7:883–92. doi: 10.1016/S2215-0366(20)30308-4, 32707037 PMC7373389

[41] Engwicht E Petersen J Braehler E Wicke F Koenig J Muenzel T . Gendered analysis of care work burden and mental health using data from the Gutenberg Covid-19 study. Sci Rep. (2025) 15:26686. doi: 10.1038/s41598-025-11841-x PMC12284194 40695939

[42] Adams-Prassl A Boneva T Golin M Rauh C. The impact of the coronavirus lockdown on mental health: evidence from the United States. Econ Policy. (2022) 37:139–55. doi: 10.1093/epolic/eiac002

[43] Beutel ME Hettich N Ernst M Schmutzer G Tibubos AN Braehler E. Mental health and loneliness in the German general population during the COVID-19 pandemic compared to a representative pre-pandemic assessment. Sci Rep. (2021) 11:14946. doi: 10.1038/s41598-021-94434-8, 34294816 PMC8298418

[44] Pieh C Budimir S Probst T. The effect of age, gender, income, work, and physical activity on mental health during coronavirus disease (COVID-19) lockdown in Austria. J Psychosom Res. (2020) 136:110186. doi: 10.1016/j.jpsychores.2020.110186, 32682159 PMC7832650

[45] Daly M Sutin AR Robinson E. Longitudinal changes in mental health and the COVID-19 pandemic: evidence from the UK household longitudinal study. Psychol Med. (2022) 52:2549–58. doi: 10.1017/S0033291720004432, 33183370 PMC7737138

[46] Zheng F Li C Hua R Liang J Gao D Xie W. Sex differences in changes of depressive symptoms among older adults before and during the COVID-19 pandemic: evidence from two longitudinal cohorts. BMC Geriatr. (2023) 23:64. doi: 10.1186/s12877-023-03744-1, 36726098 PMC9891753

[47] UN Women. (2021). Report on the UN Women global response to COVID-19. New York. Available online at: https://www.unwomen.org/sites/default/files/Headquarters/Attachments/Sections/Library/Publications/2021/Report-on-the-UN-Women-global-response-to-COVID-19-en.pdf (Accessed September 8, 2025).

[48] Morgan R Tan H-L Oveisi N Memmott C Korzuchowski A Hawkins K . Women healthcare workers' experiences during COVID-19 and other crises: a scoping review. Int J Nurs Stud Adv. (2022) 4:100066. doi: 10.1016/j.ijnsa.2022.100066, 35128472 PMC8801061

[49] Power K. The COVID-19 pandemic has increased the care burden of women and families. Sustain Sci Pract Policy. (2020) 16:67–73. doi: 10.1080/15487733.2020.1776561

[50] Andrew A. Cattan S. Costa Dias M. Farquharson C. Kraftman L. Krutikova S. (2020). How are mothers and fathers balancing work and family under lockdown Institute for Fiscal Studies. Available online at: https://web.archive.org/web/20200921071010id_/https://www.ifs.org.uk/uploads/BN290-Mothers-and-fathers-balancing-work-and-life-under-lockdown.pdf (Accessed September 5, 2025).

[51] Sardella A Lenzo V Basile G Musetti A Franceschini C Quattropani MC. Gender and psychosocial differences in psychological resilience among a community of older adults during the COVID-19 pandemic. J Pers Med. (2022) 12:1414. doi: 10.3390/jpm12091414 PMC9504613 36143198

[52] Beutel ME Klein EM Brähler E Reiner I Jünger C Michal M . Loneliness in the general population: prevalence, determinants and relations to mental health. BMC Psychiatry. (2017) 17:1–7. doi: 10.1186/s12888-017-1262-x, 28320380 PMC5359916

[53] Cacioppo JT Hughes ME Waite LJ Hawkley LC Thisted RA. Loneliness as a specific risk factor for depressive symptoms: cross-sectional and longitudinal analyses. Psychol Aging. (2006) 21:140–51. doi: 10.1037/0882-7974.21.1.140, 16594799

[54] Steen OD Ori APS Wardenaar KJ van Loo HM. Loneliness associates strongly with anxiety and depression during the COVID pandemic, especially in men and younger adults. Sci Rep. (2022) 12:9517. doi: 10.1038/s41598-022-13049-9, 35681066 PMC9178936

