# Supplementary material for: Vocational factors and mental health during the COVID-19 pandemic in middle-aged and older adults: longitudinal evidence from a population-based cohort
Source: Front Public Health. 2026 Sep 11;14:1803297. doi: 10.3389/fpubh.2026.1803297 (PMC13613494; doi:10.3389/fpubh.2026.1803297)
Supplement: Supplementary file 1 [file Table_1.docx]

**Supplementary Table 1.** Overview of all ANCOVAs performed to identify groups particularly affected by the pandemic with a significant difference but a very small effect.

| **PHQ-9** | **p** | ***Cohen´s d*** | **Post-hoc** |
| --- | --- | --- | --- |
| Minors in the household | *** | 0.14 | minors in the household * no minors in the household |
| Change in net income since the pandemic | *** | 0.13 | no change * reduced |
| Home office | *** | 0.13 | some home office but not all the time * only in the company |
| Government funds | *** | 0.09 | yes * no |
| for self-employed: business closes | ** | 0.06 | yes * no |
| **GAD-2** | **p** | ***Cohen´s d*** | **Post-hoc** |
| Relationship status | *** | 0.11 | Yes, separated household* yes, joint households / no relationship |
| Employment status | *** | 0.11 | retired * employed |
| Change in net income since the pandemic | *** | 0.11 | no change * reduced |
| Government funds | *** | 0.11 | yes * no |

Notes: * indicate post-hoc compared categories; underlined variables show lower PHQ-9 / GAD-2 values.

**Supplementary Table 2.** Comparison of ANCOVA-derived effect sizes across separate T2 and T3 analyses and the main analysis using averaged T2/T3 outcomes.

| **PHQ-9** | **T2 F(df), p, d** | **T3 F(df), p, d** | **Main analysis (T2/T3 mean) d** | **pattern** |
| --- | --- | --- | --- | --- |
| Age group | 17.83 (4; 5474), p < .001,  d = 0.23 | 19.87 (4; 5611), p < .001,  d = 0.24 | 0.24 | Similar direction/effect |
| Sex | 62,18 (1; 5810), p < .001,  d = 0.21 | 53,56 (1; 5614), p < .001,  d = 0.19 | 0.21 | Similar direction/effect |
| Increase in weekly working hours | 28.02 (1; 3549), p < .001,  d = 0.18 | 28.39 (1; 3289), p < .001,  d = 0.19 | 0.20 | Similar direction/effect |
| Volume of employment | 18.20 (3; 5269), p < .001,  d = 0.20 | 13.34 (3; 5494), p < .001,  d = 0.17 | 0.19 | Similar direction/effect |
| Employment status | 11.81 (4; 5208), p < .001,  d = 0.19 | 9.32 (4; 4846), p < .001,  d = 0.18 | 0.18 | Similar direction/effect |
| Relationship status | 15.64 (2; 4562), p < .001,  d = 0.17 | 14.08 (2; 4241), p < .001,  d = 0.17 | 0.17 | Similar direction/effect |
| Occupational groups | 4.59 (2; 3055), p = .003,  d = 0.13 | 6.69 (2; 2831), p < .001,  d = 0.17 | 0.16 | Similar direction/effect |
| **GAD-2** | **T2 F(df), p, d** | **T3 F(df), p, d** | **Main analysis (T2/T3 mean) d** | **pattern** |
| Sex | 39.66 (1; 4606), p < .001,  d = 0.19 | 63,75 (1; 6075), p < .001,  d = 0.20 | 0.21 | Similar direction/effect |
| Volume of employment | 10.30 (3; 4141), p < .001,  d = 0.17 | 13.30 (3; 5937), p < .001,  d = 0.17 | 0.18 | Similar direction/effect |
| Increase in weekly working hours | 16.38 (1; 2823), p < .001,  d = 0.16 | 19.01 (1; 3500), p < .001,  d = 0.14 | 0.17 | Similar direction/effect |
| Age group | 4.20 (4; 4291), p =.002,  d = 0.13 | 10.30 (4; 6072), p < .001,  d = 0.17 | 0.16 | Similar direction/effect |

Note: The T2/T3 mean column refers to the main analysis using the averaged T2/T3 outcome and should not be interpreted as the arithmetic mean of the T2 and T3 effect sizes. Cohen’s d values were derived from partial eta squared and should therefore be interpreted as approximate d-equivalent effect sizes, particularly for predictors with more than two categories.
